# In vitro dexamethasone treatment does not induce alternative *ATM* transcripts in cells from Ataxia–Telangiectasia patients

**DOI:** 10.1038/s41598-020-77352-z

**Published:** 2020-11-19

**Authors:** Elisa Pozzi, Elisa Giorgio, Cecilia Mancini, Nicola Lo Buono, Stefania Augeri, Marta Ferrero, Eleonora Di Gregorio, Evelise Riberi, Maria Vinciguerra, Lorenzo Nanetti, Federico Tommaso Bianchi, Maria Paola Sassi, Vincenzo Costanzo, Caterina Mariotti, Ada Funaro, Simona Cavalieri, Alfredo Brusco

**Affiliations:** 1 Department of Medical Sciences, University of Torino, via Santena 19, 10126 Turin, Italy; 2 Laboratory of Immune-Mediated Diseases, San Raffaele Diabetes Research Institute (DRI), 20132 Milan, Italy; 3 Unit of Medical Genetics, “Città Della Salute E Della Scienza” University Hospital, 10126 Turin, Italy; 4 Department of Public Health and Pediatrics, University of Torino, 10126 Turin, Italy; 5 DNA Metabolism Laboratory, FIRC Institute of Molecular Oncology (IFOM), 20139 Milan, Italy; 6 Unit of Genetics of Neurodegenerative and Metabolic Diseases, Fondazione IRCCS Istituto Neurologico “Carlo Besta”, 20133 Milan, Italy; 7 Department of Molecular Biotechnologies and Health Sciences, Neuroscience Institute Cavalieri Ottolenghi, 10043 Orbassano, TO Italy; 8 Istituto Nazionale di RIcerca Metrologica INRIM, 10135 Turin, Italy

**Keywords:** Gene regulation, Experimental models of disease, Molecular medicine

## Abstract

Short term treatment with low doses of glucocorticoid analogues has been shown to ameliorate neurological symptoms in Ataxia–Telangiectasia (A–T), a rare autosomal recessive multisystem disease that mainly affects the cerebellum, immune system, and lungs. Molecular mechanisms underlying this clinical observation are unclear. We aimed at evaluating the effect of dexamethasone on the induction of alternative *ATM* transcripts (*ATMdexa1*). We showed that dexamethasone cannot induce an alternative *ATM* transcript in control and A–T lymphoblasts and primary fibroblasts, or in an *ATM*-*knockout* HeLa cell line. We also demonstrated that some of the reported readouts associated with *ATMdexa1* are due to cellular artifacts and the direct induction of γH2AX by dexamethasone via DNA-PK*.* Finally, we suggest caution in interpreting dexamethasone effects in vitro for the results to be translated into a rational use of the drug in A–T patients.

## Introduction

Ataxia–Telangiectasia (A–T; MIM#208900) is a rare autosomal recessive multisystem disorder caused by biallelic pathogenic variants in the *ATM* gene (MIM#607585). Most of these variants are null changes leading to a complete loss of ATM protein function. A–T patients show early-onset progressive cerebellar neurodegeneration, oculocutaneous telangiectasias, immunodeficiency and a high incidence of infections and cancers^1–3^. In classic A–T, patients are wheelchair-dependent by the age of 10 years and their life expectancy is approximately 25 years^4–6^.

*ATM* encodes a large 3056 amino acid protein whose main role is coordinating the cellular response to DNA double strand breaks^7^, but it is also involved in the response to oxidative stress, and other forms of genotoxic stress. ATM is active in cell signaling pathways involved in maintaining cellular homeostasis, and is known to directly phosphorylate and regulate a list of several hundreds of substrates^7–12^.

Since ATM is ubiquitously expressed, the reason why cerebellar Purkinje cells are so incredibly sensitive to its loss, while other neurons are unaffected, is still unknown.

No effective disease-modifying therapy is presently available for A–T; however, in recent years, some studies have demonstrated that short-term treatments with low doses of glucocorticoids ameliorate the neurological symptoms of A–T patients without relevant side effects^13–17^. After an observational study which suggested that betamethasone may improve neurologic functions in patients with A–T^13^, short-term trials confirmed the efficacy of oral betamethasone^14^. In particular, speech disturbance and stance, as well as the quality of motor coordination, were the most sensitive neurological parameters^14, 15, 18^. A recent study suggested that a daily dose of 0.005 mg/kg betamethasone, is effective in some A–T patients, and can be considered for occasional usage under medical supervision. However, the long-term side-effects versus efficacy of this treatment has not yet been evaluated^17^.

Other research groups showed that infusions of autologous erythrocytes loaded with dexamethasone were effective in improving neurological symptoms in some A–T patients^19^. This procedure takes advantage of an autologous erythrocyte-based drug delivery system, and is currently used in an international, multi-centre, randomized, prospective, double-blind, placebo-controlled, phase III study (https://clinicaltrials.gov/ct2/show/NCT02770807). The procedure is invasive, requiring monthly blood samples of 50 ml and subsequent transfusions^19^.

The cellular/molecular mechanism(s) underlying the glucocorticoid clinical effects in A–T are currently unclear. Response to treatment occurs within hours, and incoordination rapidly reoccurs upon suspension of the drug. A–T patients, who exhibited a good motor response to betamethasone treatment had increased activation in relevant cortical areas has been reported, suggesting that glucocorticoids may facilitate cortical compensatory mechanisms on cerebellar dysfunction^20^.

**Figure 1:**
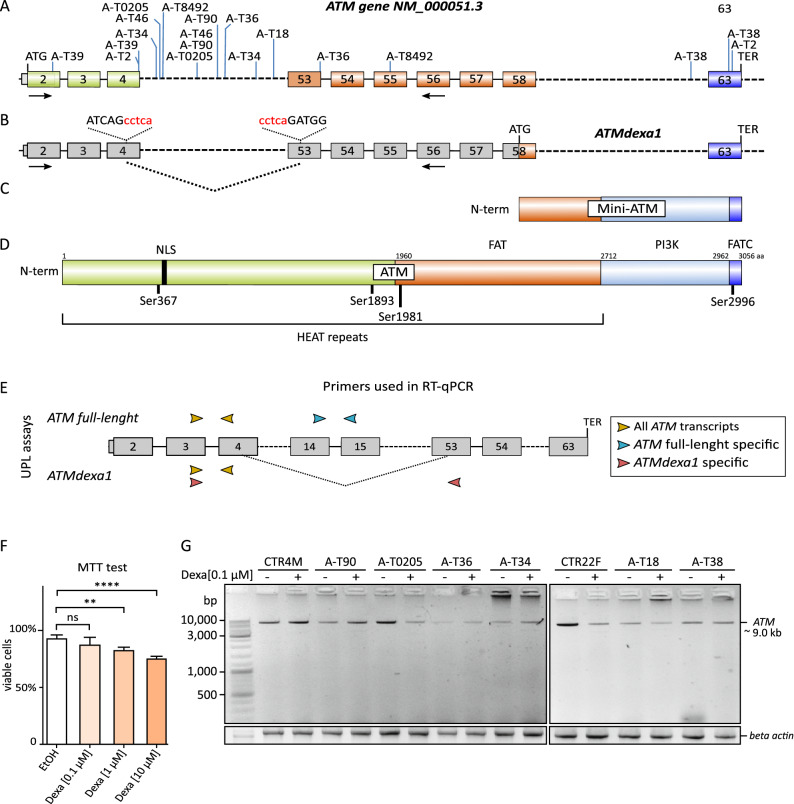
*ATM* and *ATMdexa1* transcripts and their encoded proteins; MTT assay and cDNA analysis. (**A**) Full-length *ATM* transcript (NM_000051.3) with the first ATG (methionine) and termination codon (TER). The approximate position of the pathogenic variants in the A–T patients used in this study are indicated. (**B**) The *ATMdexa1* variant transcript was previously reported by^21^. No GT-AG canonical intronic consensus splicing sequences are present at the exon 4 and 53 boundaries; hence, an uncommon splicing event should occur between two internal sequences within these exons. Both exon 4 and exon 53 breakpoints in *ATMdexa1* are reported to be flanked by a “cctca” sequence (in red). The first ATG within exon 58 and the termination codon in exon 63 (TER) are indicated. Arrows in exon 2 and exon 56 indicate forward and reverse primers used for *ATM* cDNA analysis (Fig. 2C). The encoded proteins of the two transcripts are illustrated in panels (**C**) (*ATMdexa1-* > mini-ATM) and (**D**) (full length *ATM-* > ATM). The main domains of the ATM protein are coloured as such: FAT (FRAP, ATM and TRRAP; a.a. 1960–2566 orange), PI3 kinase-domain (PI3K; a.a. 2712–2962 light blue), and FATC (FRAP, ATM and TRRAP C-terminal; a.a. 2963–3056 blue). The positions of auto-phosphorylation sites in ATM are indicated (Ser367, Ser1893, Ser1981 and Ser2996). (**E**) Schematic representation of the primers used for RT-qPCR assays. Primers mapping on exons 3 (forward) and 4 (reverse) (yellow arrows) can detect both full-length *ATM* and *ATMdexa1*. Primers mapping on exon 14 (forward) and 15 (reverse) can amplify full-length *ATM* but not *ATMdexa1* (blue arrows). Primers mapping on exon 3 (forward) and 53 (reverse) should amplify *ATMdexa1* and are unable to amplify full-length *ATM.* (**F**) MTT cell viability assay performed on control cell lines after treatment with dexamethasone. LCLs were treated with dexamethasone for 24 h at different concentrations of the drug (0.1 μM, 1 μM and 10 μM). As a control, cells were cultured with 0.1% EtOH. Histograms display the percentage of viable cells after treatment. The MTT assay data were analysed using the Graph Pad Prism 6.0 software, one-way ANOVA followed by the Bonferroni post hoc test (*p < 0.05, **p < 0.01, ***p < 0.001 vs. control cells; *ns* not significant). G. *ATM* transcripts visualization by long-range PCR and agarose gel in six A–T (variants are indicated in Fig. 1A and Supplemental Table 1) and two control (CTR) LCLs did not reveal any alternative transcript with or without 72 h treatment with dexamethasone (0.1 µM).

An explanation for the corticosteroid response in A–T patients was the description of dexamethasone induction of non-canonical *ATM* splicing events in A–T cell lines. Dexamethasone can allow the synthesis of a non-canonical *ATM* transcript (*ATMdexa1*) and protein (mini-ATM), with some of the full-length ATM functions (Fig. 1A–D).

Here, we investigated this *ATM* splicing event using B-lymphoblastoid cell lines (LCLs) and fibroblasts from A–T patients with different *ATM* pathogenic variants and a HeLa CRISPR/*ATM*-*knockout* cell line model without finding any evidence of *ATMdexa1* or other splicing anomalies induced by dexamethasone.

## Materials and methods

### Cell lines

A–T (Supplemental Table 1) and control LCLs were established from fresh lymphocytes infected by Epstein-Barr virus and maintained in RPMI-1640 medium (Sigma Aldrich, Italy) supplemented with 2 mMol Glutamine, 50 U/ml penicillin, 50 µg/ml streptomycin and 10% Fetal Bovine Serum (FBS, Gibco, Thermo Fisher Scientifics, Waltham, MA, USA).

A–T and control human primary fibroblasts were obtained from skin biopsies after overnight incubation in Dulbecco’s Modified Eagle Medium (DMEM, Sigma Aldrich, Italy) with 10% FBS with collagenase (160 µg/ml) and then cultured in DMEM with 2 mMol Glutamine, 50 U/ml penicillin, 50 µg/ml streptomycin, 1 mMol Sodium Pyruvate and 10% FBS. Cells from passages 5 to 9 were used for all experiments, and cells from A–T patients and healthy controls at the same passage were compared.

HeLa CRISPR/*ATM*-*knockout* were generated using the CRISPR/Cas9 method and maintained in Minimum Essential Medium (Biowest #L0440), supplemented with 10% FBS, 2 mMol Glutamine, 1 mMol Sodium Pyruvate, 0.1 mMol non-essential amino acids (NEAA), 50 U/ml penicillin, and 50 µg/ml streptomycin. All cells were maintained at 37 °C with 5% CO_2_. Informed consent was obtained from participants for the use of blood and skin samples. The study was approved by the institutional Internal Review Board of the Department of Medical Sciences, University of Torino, and C. Besta Neurological Institute. Methods were carried out in accordance with the relevant guidelines and regulations. Dexamethasone doses and times (0.1 μM for 24 h and 72 h) used to analyse mRNA and protein expression were in accordance with published experimental conditions^21^.

### *ATM* transcript and expression analysis

Total RNA was extracted from 5 × 10^6^ LCLs and from 2 × 10^5^ fibroblasts (from six A–T patients and four healthy controls) and from 2 × 10^5^ HeLa CRISPR/*ATM*-*knockout* cells using the Direct-zol™ Kits- RNA extraction kit (Zymo Research, Irvine, California, USA) according to the manufacturer's instructions. Retrotranscription of 1 µg RNA was carried out using the M-MLV Reverse Transcriptase following the manufacturer’s instructions (Thermo Fisher Scientific, Waltham, Massachusetts, USA). Long range PCR for the *ATM* transcript (exons 2–56; reference *ATM* sequence NM_000051.3) was performed in a total volume of 50 µl with a final concentration of 200 nM of each primer (Supplemental Table 2), 400 μM of dNTPs, 1 × enzyme buffer and 2.5 units of LA polymerase (Takara Bio Inc., Otsu, Shiga 520–2193, Japan), using the following cycling parameters: 1 min at 94 °C, followed by 30 cycles of 10 s at 98 °C, and 11 min/kb at the annealing temperature, with a final extension at 72 °C for 10 min. *Beta-actin* was amplified as a control in a final volume of 25 µl with a final concentration of 500 nM of each primer, 200 µM of dNTPs, 1 × KAPA2G enzyme buffer and 0.5 units of KAPA2G Fast HotStart polymerase (KAPA Biosystems, Wilmington, MA, USA), under the following cycling parameters: 3 min at 95 °C, followed by 25 cycles of 15 s at 95 °C, and 15 s at 60 °C and 15 s at 72 °C followed by a final extension at 72 °C for 1 min. Amplification products were separated on a 0.6% or 1% TBE-agarose gel for *ATM* and *β-actin,* respectively, then stained with 1X Midori Green DNA stain (Nippon Genetics Europe Gmbh) and visualized using a GelDOC apparatus (Biorad, Hercules, California, United States).

### Absolute quantification by RT-qPCR

For an absolute quantification of full-length *ATM* and *ATMdexa1* transcripts, we designed three different reverse-transcription quantitative real-time PCR (RT-qPCR) assays using the Universal Probe Library method (UPL, Roche, Mannheim, Germany), namely (1) an *ATMdexa1*-specific assay (primers on cDNA spanning the exons 3–53 junction); (2) a full-length *ATM* specific assay (primers on cDNA spanning the exons 14–15 junction); and (3) an *ATM* assay able to detect both full length and *ATMdexa1* (primers on cDNA spanning the exons 3–4 junction) (Fig. 1E). Amplifications were carried out on an ABI-Prism 7500 Fast instrument, using the ABI 2X TaqMan Gene Expression master mix, according to the manufacturer’s instructions (Applied Biosystems, Thermo Fisher Scientific).

To obtain an absolute quantification, we generated three calibration curves, exploiting two plasmid vectors containing either the full-length *ATM* coding sequence (pMAT plasmid^22^) or an artificial *ATMdexa1* amplimer (pGEM-ATMdexa1 plasmid). This was generated by the overlap-extension method described in Supplemental Fig. 1. Each plasmid was prepared at an initial concentration of 1 × 10^6^ copies/µL and a dilution series was prepared (#copies/µL: 10^3^, 5 × 10^3^, 10^4^, 5 × 10^4^, 10^5^, 5 × 10^5^). Each dilution was performed in triplicate and the mean was used as a reference to calculate the calibration curve (Supplemental Table 3). Three A–T and control human LCLs treated with vehicle (EtOH) or with 0.1 µM dexamethasone were tested using the above UPL assays and the number of mRNA copies was inferred interpolating the Ct values to standard curves (Supplemental Table 4 in dataset 1).

### Western blotting

Total protein lysates were extracted from LCLs and primary fibroblasts of six A–T patients and four healthy controls, using RIPA Lysis Buffer [Tris–Cl (50 mM, pH 7.5), NaCl (150 mM), NP40 (1%), Na Deoxycholate (0.5%), DTT (0.1 M), EDTA (5 mM), HaltTM Protease and Phosphatase inhibitor cocktail (Thermo Fisher Scientific)]. Nuclear lysates were extracted from fibroblasts using NE-PER Nuclear and Cytoplasmic Extraction Kit following the manufacturer's protocol (Thermo Fisher Scientific). Protein quantification was measured using the Bradford assay (Bio-Rad) according to the manufacturer’s protocol. Protein lysates (20 µg) were denatured for 10 min at 70 °C in LaemmLi Sample Buffer (4% SDS, 20% glycerol, 10% 2-mercaptoethanol, 0.004% bromophenol blue and 125 mM Tris HCl, pH 6.8) and reducing agent (50 mM dithiothreitol, DTT), resolved by SDS–PAGE (4–12%), using Tris–Glycine-SDS buffer (Thermo Fisher Scientific) and electro-transferred onto nitrocellulose membranes (Bio-Rad) for 1 hr at 125 V. Membranes were blocked with TBS-T [50 mM Tris and 150 mM NaCl, pH 7.6, and 0.1% Tween-20 (Sigma, Italy)] with 5% BSA and probed with primary antibodies against ATM (2C1) (1:400; Ab sc. 23921, Santa Cruz Biotechnology, Italy), p-ATM (1:5000; Ab Cat. No. 32420, Abcam, Italy), γH2AX (1:3000; Ab Cat. No. NB100-384, Novus, Europe). β-Actin (1:2000; Cat. No. NB600-532H, Novus, Europe) or Vinculin (1:2000; Cat. No. AB6039, Merck Millipore, Italy) antibodies were used as loading controls. For western blot analyses, cells were incubated for 72 h in RPMI or DMEM medium supplemented with 5% FBS, before lysis for extraction of total or nuclear proteins. Band density was quantified by densitometric analysis using the Image Lab 3.0 software (Bio-Rad).

LCLs and fibroblasts were irradiated at a dose rate of 10 Gy/min at a distance of 40 cm (Radgil, Gilardoni Instruments, Italy). Inhibition of DNA-PK kinase activity was performed by adding 10 μM NU7441 (Sigma Aldrich) for 1 h to untreated cells or cells pre-treated with 0.1 μM dexamethasone for 72 h before irradiation (IR). Cells were then incubated at 37 °C for 1 h before nuclear protein extraction. Drugs were maintained in growth medium until time of harvest.

### Statistical analyses

Experiments were performed in triplicate and repeated at least twice, unless otherwise specified. Values are given as means and standard deviations, or as fold-changes. Mean values of variables with a normal distribution were reported, and comparisons between control groups and patient groups were conducted using the Student's *t* test. When the distribution of data was not normal (densitometry analyses of all western blots), variables were presented as median values, and differences between two groups were calculated using the Mann–Whitney test. Significance of gene expression and enzyme activity data was calculated using the Student's *t* test (unpaired). Statistical calculations were performed using GraphPad Statistics Software Version 6.0 (GraphPad Software, Inc., USA). *p* values of < 0.05 were considered statistically significant.

## Results and discussion

The proposed use of glucocorticoids for A–T therapy has prompted in vitro studies aimed at analysing their possible compensating role of *ATM* deficiency. Our initial aim was to unravel the effect of glucosteroids on A–T lymphoblastoid cells and primary fibroblasts, two cell types widely used as experimental models in this disease. Dexamethasone has been reported to induce *ATM* alternative splicing, resulting in the *ATMdexa1* transcript*,* which encodes a functional mini-ATM protein^21, 23^.

To validate this finding, we generated five A–T lymphoblastoid cell lines (LCLs) with *ATM* gene pathogenic variants outside the *ATMdexa1* encoding exons on one or both alleles, and one LCL (AT-38) who carried two nonsense variants in exons 63 and 65 included in *ATMdexa1* (Fig. 1A and Supplemental Table 1). To verify the effects of dexamethasone on cell viability, we performed an MTT viability assay, treating control LCLs with increasing doses of the drug (0.1, 1, or 10 µM for 24 h). Dexamethasone showed a modest but significant decrease in viability with doses ≥ 1 µM (Fig. 1F), which largely exceeds the dose of 0.1 µM for 24 h alleged to induce *ATMdexa1* in vitro. Following the previously published protocol^21^, we tested the effect of dexamethasone on *ATM* transcripts in both LCLs and fibroblasts (0.1 µM, 24 h). Using a forward primer on exon 2 and a reverse primer on exon 56 of the *ATM* cDNA (NM_00051.3), we were able to amplify the native *ATM* transcript (~ 9.0 kb) in both treated and untreated cells (Fig. 1G). No changes were observed after treatment in band intensity or in the appearance of additional bands corresponding to the size expected for *ATMdexa1* (~ 1.6 kb). Even if we increased dexamethasone by tenfold compared with the suggested protocol and enhanced gel contrast (1 µM dexamethasone for 24 h), no additional band was detectable (Supplemental Fig. 2). Analogous results were obtained in primary fibroblast cultures from six A–T patients and four healthy controls, and in a HeLa CRISPR/*ATM*-*knockout* cell line (4C18) (data not shown).

**Figure 2:**
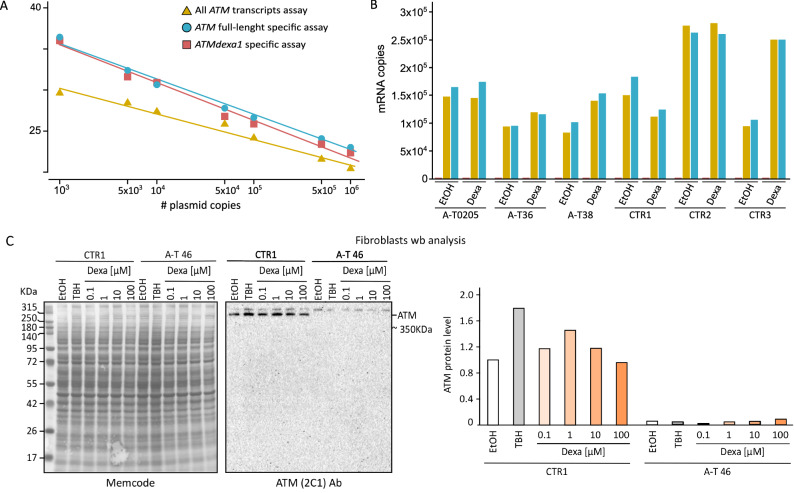
Absolute reverse-transcription quantification of *ATM* transcripts and ATM western blot. (**A**) Calibration curves for the three RT-qPCR assays shown in Fig. 1E. Y-axis reports the Ct values obtained from quantitative real time analysis; on the X-axis, the number of plasmid copies used (data in Supplemental Table 3). For full-length *ATM,* we used a plasmid containing the entire gene coding region (pMAT); for *ATMdexa1* we generated a specific plasmid as described in Supplemental Fig. 1. (**B**) Absolute PCR quantification, in number of copies, of the full-length *ATM* and *ATMdexa1* transcripts in three A–T cell lines and three controls, treated with solvent (EtOH) or dexamethasone. We did not detect *ATMdexa1* in any case (Supplemental Table 4 in dataset 1)*.* (**C**) Western blot analysis of the ATM protein in control (CTR1) and A-T46 fibroblasts; cells were treated with vehicle (EtOH), 300 μM of *t*-butyl hydroperoxide (TBH) or dexamethasone at increasing concentrations (tenfold increase from 0.1 to 100 µM). Cell lysates were analyzed by western blotting using Memcode as reference (left panel) or the ATM 2C1 antibody, specific for the C-terminal portion of the ATM protein (aa. 2577–3056). No bands beyond ATM were visible on the gel. Histograms show the quantitative analysis of ATM in CTR1 and A-T46.

To have a quantitative measure of *ATM* transcripts, and evaluate the detection limits of our technique, we set up three real-time quantification assays for *ATM* able to detect: 1) full-length *ATM* and *ATMdexa1;* 2) full-length *ATM* only transcripts; 3) *ATMdexa1* transcripts. To generate the calibration curves, we used a serial dilution from 10^3^ to 10^6^ copies of a plasmid containing full-length *ATM* (pMAT) and a plasmid containing *ATMdexa1,* generated in our laboratory (pGEM-ATMdexa1 plasmid). We were able to clearly detect as low as 10^3^ copies of both full ATM and *ATMdexa1*, although this was not the lower limit of the test (Fig. 2B). The *ATM* transcript was expressed at 63 ± 22% (median ± S.D.) in A–T cases versus controls (Fig. 2C), without any relevant differences between untreated cells or cells treated with dexamethasone, apart from the CTR3 treated cell line (Fig. 2C). We were unable to detect *ATMdexa1* in any sample (Fig. 2C). Notably, there was no difference between the number of cDNA copies estimated by the full-length *ATM* assay, which includes all *ATM* transcripts*,* and the full-length *ATM* specific assay, which does not include *ATMdexa1*. This further corroborates the absence of an *ATMdexa1* transcript.

Using the 2C1 antibody, raised against the C-terminal portion of the ATM protein (aa. 2577–3056), we were able to reveal a unique band corresponding to the native ATM in control fibroblasts but not in A-T fibroblasts. No additional proteins were evident, further proving the absence of mini-ATM (Fig. 2C).

Since *ATMdexa1* was reported in both stimulated and non-stimulated cells, we searched available databases (UCSC, ENSEMBL) to have *in-silico* evidence of the *ATMdexa1* transcript. *ATM* had a single validated protein-encoding transcript (NM_000051.3), and some shorter transcripts, none of which had any similarity with the *ATMdexa1.*

A previous study^21^, also raised unanswered questions on how *ATMdexa1* is formed: (1) an atypical splicing event should take place between exons 4 and 53, without any canonical splicing site. The authors state this can happen by a rare SDR-splicing, reported only once in *Oryza sativa*, and never described in Metazoa^24^; (2) the generation of the “mini-ATM” protein starts from a non-canonical ATG-encoding first methionine within exon 58 (Met 2806), in the absence of a translational-initiation consensus. This ATG would produce an ATM variant protein beginning from codon 8418^21^, and lacking the N-terminal, which carries nuclear localization sequences, several critical phosphorylation sites, and binding sites for chromatin and ATM-interacting proteins. Published data are against the notion that such a protein is functional, at least for its nuclear activity. ATM without the N-terminus is unable to fully localize to the nucleus and therefore activate DNA repair effectors^7, 25–28^.

**Figure 3:**
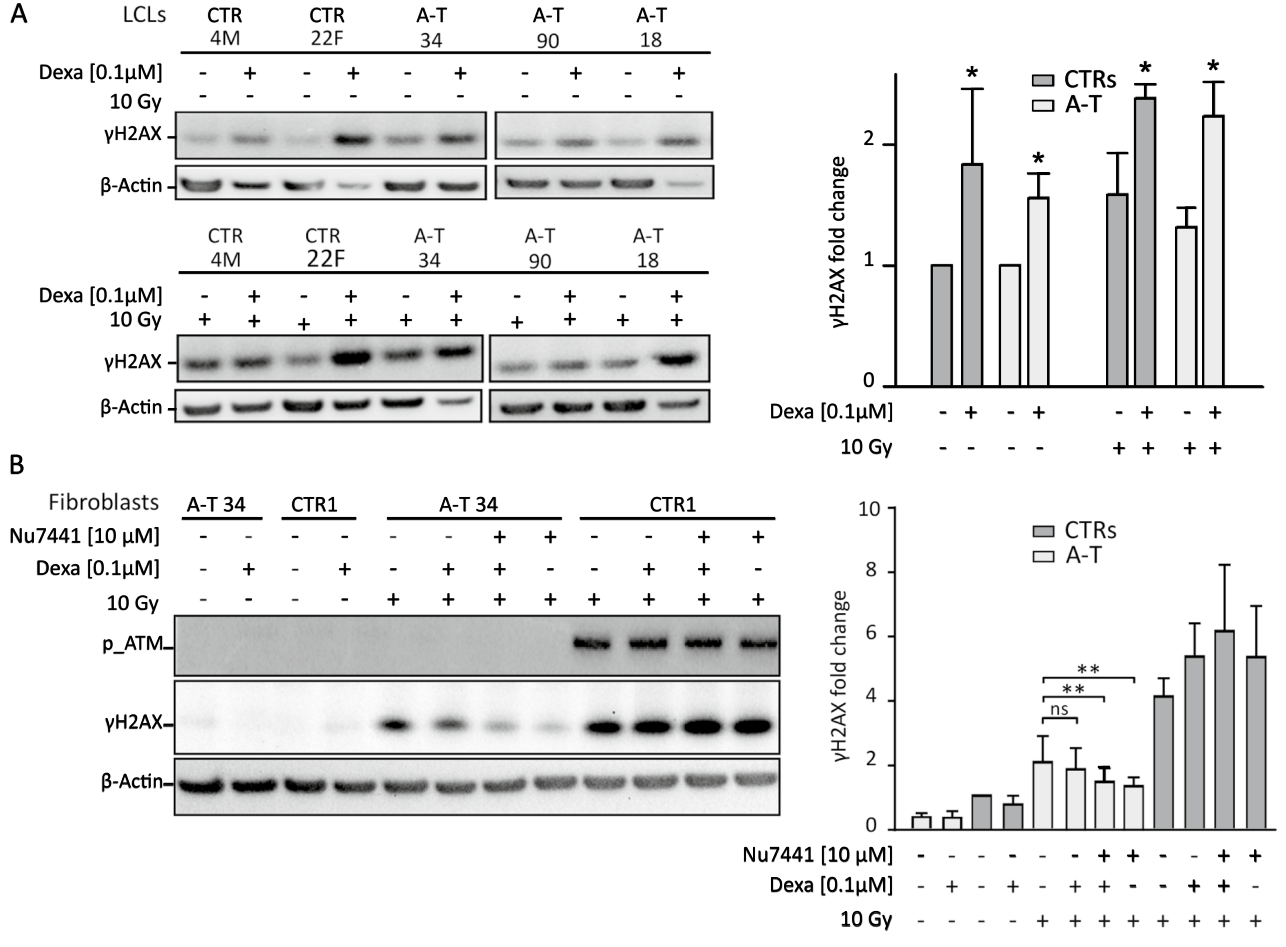
Analysis of the activity of ATM substrates activity. (**A**) Western blot analysis of H2AX Ser-139 phosphorylation (γH2AX) on nuclear extracts from LCLs. Dexa 0.1 µM indicates dexamethasone treatment for 72 h; 10 Gy = 10-grey ionizing radiation (IR). Cells were harvested at 1 h after IR. Histograms on the right represent the densitometry analysis (mean ± SE; n = 3); fold-change was normalized on untreated control samples. (**B**) Western blot analysis shows γH2AX levels in nuclear extracts of fibroblasts after dexamethasone treatment and/or IR stimulation in the presence or absence of 10 µM NU7441, an inhibitor of DNA-PK kinase activity (1 h before IR). Inhibition of DNA-PK by NU7441(1 h before IR) reduces γH2AX, showing a role for DNA-PK in this pathway. Cells were harvested 1 h after IR. A representative western blot from patient A-T34 is shown (see also Supplemental Fig. 3). ATM phosphorylation measured using p-ATM Ab demonstrates the ATM activation in control cells. Histograms are representative of three independent experiments performed with two A–T patients’ (A-T34 and A-T46) and two control fibroblasts; fold-change was normalized on untreated control samples. (**p < 0.01; *ns* not significant).

To assess if dexamethasone influences known ATM-pathway substrates, in the absence of ATM itself, we measured the phosphorylation of H2AX at Ser139 (γH2AX), a sensitive marker of DNA damage responses^29^. We analyzed the expression of γH2AX in our A–T LCLs, both at a basal level and after 72 h of treatment with 0.1 µM dexamethasone. We found a basal phosphorylation of H2AX in both A–T and control LCLs, which increased by ~ 30–40% after dexamethasone treatment. Similar results were obtained after DNA double-strand break (DSB) induction by ionizing radiation (IR), with an additive effect of dexamethasone and IR combined treatment (Fig. 3A).

This result suggests that LCLs that are EBV-transformed cell lines are not a reliable cellular model to study dexamethasone effects^30^. Our data suggest a possible role of dexamethasone in activating H2AX, regardless of DSB damage, and explain part of the results attributed to *ATMdexa1*^21^. Histone H2AX is a substrate of several phosphoinositide 3-kinase-related protein kinases (PIKKs): besides ATM, ATR (ATM and Rad3-related) phosphorylates H2AX in response to single-stranded DNA breaks and during replication stress^31–34^, and DNA-PK (DNA-dependent protein kinase) mediates phosphorylation of H2AX in cells under hypertonic conditions and during apoptotic DNA fragmentation^24, 25, 35, 36^. Finally, several reports in the literature have shown that LCLs have such an inter-experimental variability that it can be concluded that they are an unsuitable model for DNA repair studies^30, 37, 38^.

We therefore decided to analyze H2AX activation in response to dexamethasone in primary cells, such as fibroblasts, in which basal γH2AX was almost undetectable (Fig. 3B). After irradiation, H2AX was phosphorylated in control fibroblasts and, to a lesser extent, in A–T fibroblasts, as expected, due to the absence of the ATM protein in A–T fibroblasts. We noticed dexamethasone did not increase γH2AX, either alone or in combination with IR (Fig. 3B).

Experimental data from other groups reported that DNA-PK can vicariate ATM in the DNA damage response^39–42^. Hence, we assessed the role of DNA-PK in H2AX activation using NU7441, a specific DNA-PK inhibitor. H2AX phosphorylation was reduced in irradiated A–T fibroblasts treated with NU7441 (Fig. 3B; *p* < 0.01; ~ 30% decrease; Supplemental Fig. 2). In this experimental condition, dexamethasone treatment did not affect γH2AX levels. Dexamethasone can only increase γH2AX levels in LCLs, but not in A–T and control fibroblasts, probably because LCLs undergo significant transformations to become immortal, which can alter the biology of the cell^37^.

In conclusion, our data and literature do not support the effect of dexamethasone on inducing *ATM* alternative transcripts. We reiterate that LCLs are not a suitable model to study H2AX activation, possibly due to their rapid replication. Taken together, our results suggest alternative explanations to *ATMdexa1* must be considered in interpreting the in vivo effects of dexamethasone in A–T treatment.

## Supplementary information

Supplementary Information 1.

Supplementary Information 2.

## Data Availability

All scientific data are available upon request.
