# Supplementary material for: In vitro dexamethasone treatment does not induce alternative ATM transcripts in cells from Ataxia–Telangiectasia patients
Source: Sci Rep. 2020 Nov 19;10:20182. doi: 10.1038/s41598-020-77352-z (PMC7677391; doi:10.1038/s41598-020-77352-z)
Supplement: Supplementary file 1 — Supplementary Information 1. [file 41598_2020_77352_MOESM1_ESM.pdf]

In vitro dexamethasone treatment does not induce alternative *ATM* transcripts in cells from Ataxia-Telangiectasia patients.

Elisa Pozzi1, Elisa Giorgio1, Cecilia Mancini1, Nicola Lo Buono2, Stefania Augeri1, Marta Ferrero1, Eleonora Di Gregorio3, Evelise Riberi4, Maria Vinciguerra5, Lorenzo Nanetti6, Federico Tommaso Bianchi7, Maria Paola Sassi8, Vincenzo Costanzo5, Caterina Mariotti6, Ada Funaro1, Simona Cavalieri1* and Alfredo Brusco1,3*

SUPPLEMENTAL MATERIAL

Supplemental figure 1

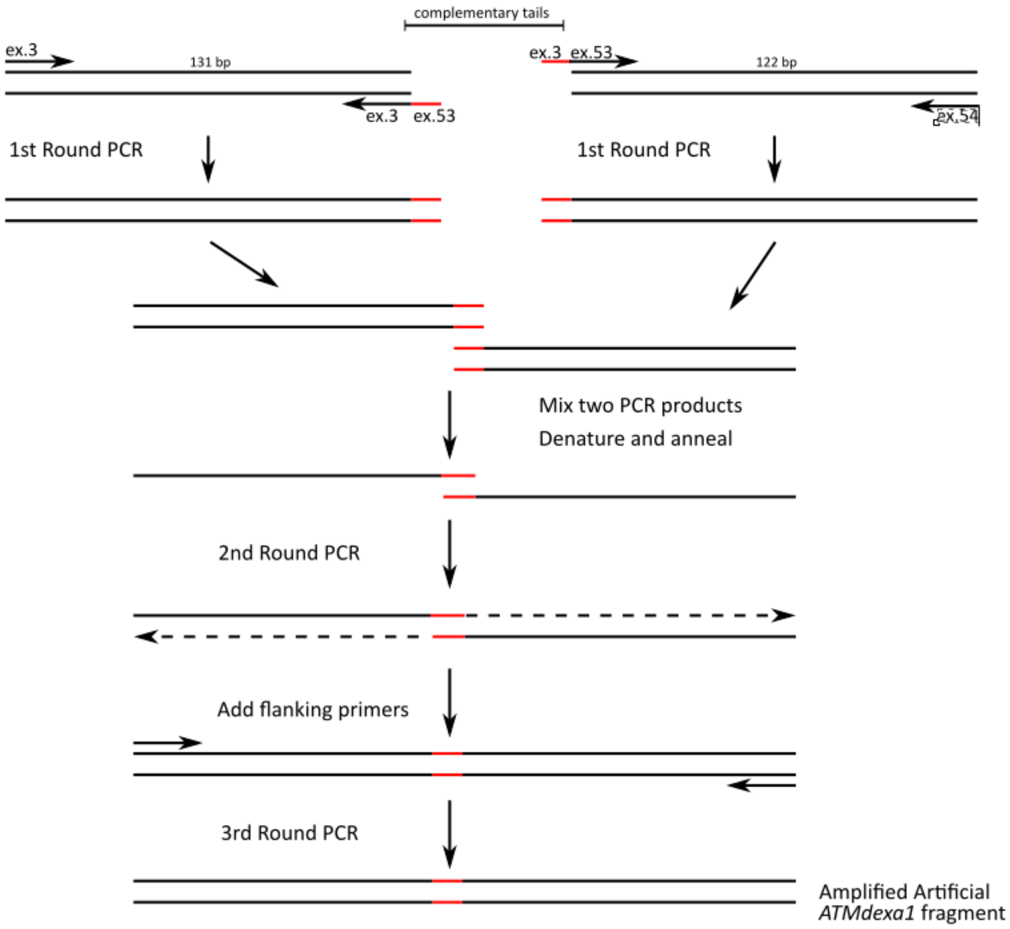

Supplemental figure 1. Schematic drawing for *ATMdexa1* artificial insert preparation.

Schematic representation of the overlap-extension method used to generate a control fragment corresponding to *ATMdexa1*. We separately amplified exons 3-53 (left amplimer) and 53-54 (right amplimer) of the *ATM* gene using primer in supplemental table 2. The reverse primers of left amplimer and the forward primer of the right amplimer contained a reverse complementary tail. In a third PCR reaction, we added a dilution of the left and right amplimers and extended to obtain a full *ATMdexa1* product. The amplimer was gel purified and cloned into the pGEM-T vector (pGEM-*ATMdexa1;* Promega, Madison, Wisconsin, USA). Clones were harvested, and the miniprep Sanger sequenced to verify the insert.

Supplemental figure 2

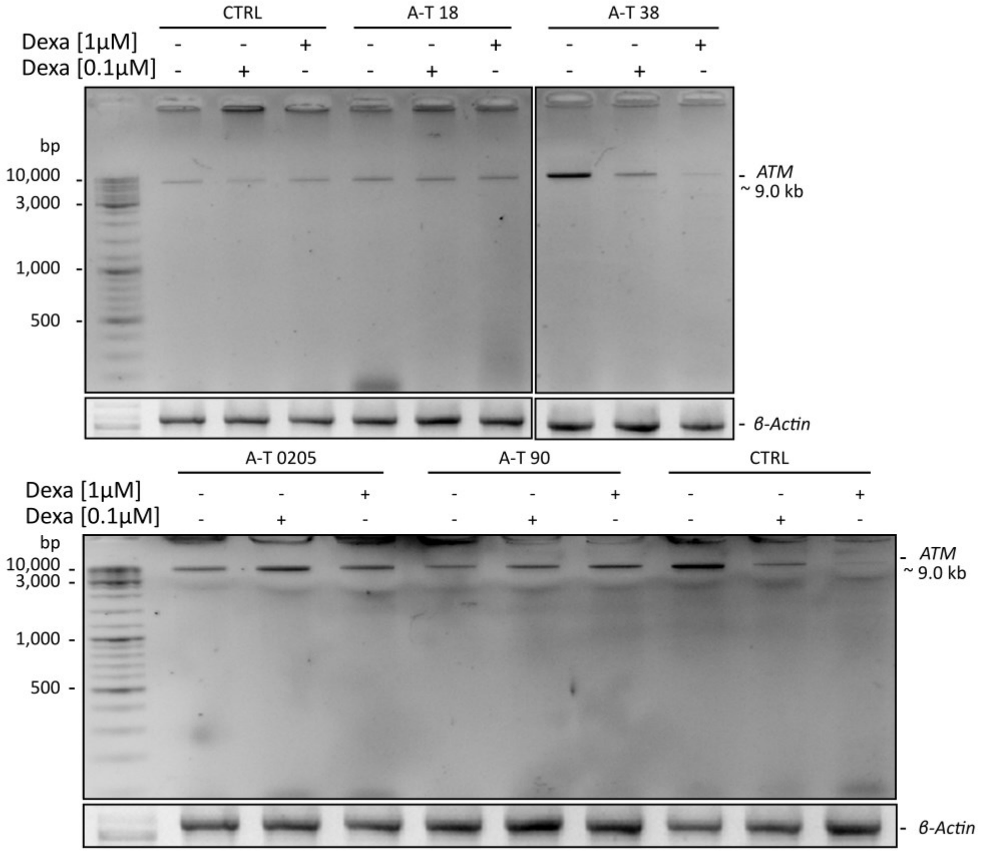

Supplemental figure 2. ***ATM*** **transcript analyses in A-T patients**

The *ATM* transcript was amplified by RT-PCR on total cDNA from four A-T and two control LCLs treated or not with 0.1 µM or 1 µM dexamethasone. No further band could be evidenced beyond the expected ~9.0-kb band, even if the dexamethasone was used 10-fold the proposed dose for revealing *ATMdexa1,* and enhancing gel contrast

Supplemental figure 3

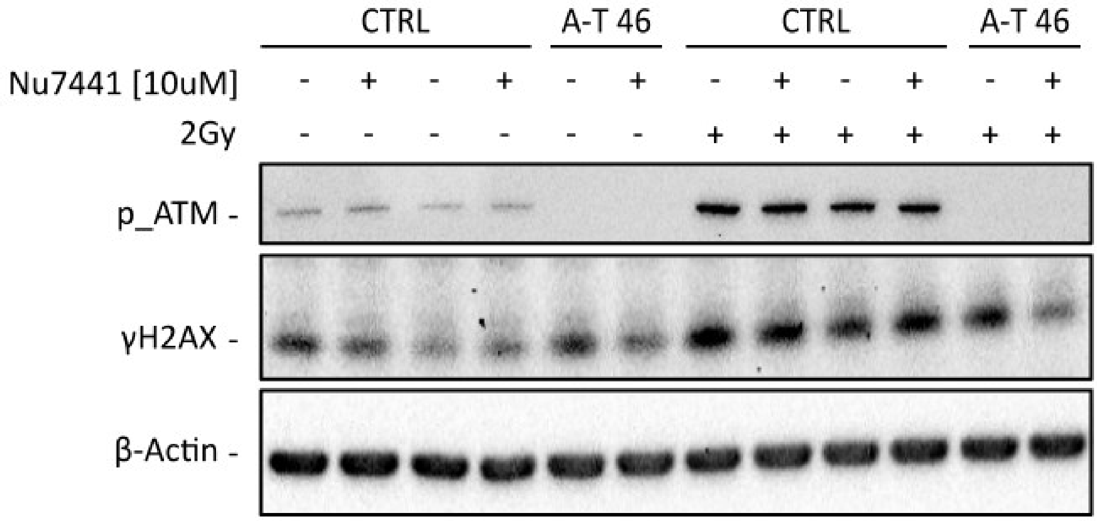

Supplemental figure 3. Analysis of *ATM* downstream effectors.

γH2AX activation after IR in A-T and control LCLs. Inhibition of DNA-PK by NU7441(1hr before IR) reduces γH2AX.

**Supplemental table 1**. *ATM* genotype (reference sequence NM_000051.3) and phenotype of A-T patients' cell lines.								
	Genotype	exon	Mutation	Consequence	ATM protein	Kinase activity	Phenotype	cell lines
AT-2	Compd Htz	4	c.331A>T	p.Arg111*	absent	no ATMp	mild	Fibro
		63	c.9103C>T	p.Leu3035Phe				
AT-18	Hom	51	c.7517_7520del GAGA	p.(Arg2506Thrfs*); p.(Arg2506Thrfs*)	absent	no ATMp	classic	LCLs
AT-34	Compd Htz	43	c.6326G>A	p.(Trp2109*)	< 10%	no ATMp	mild	LCLs; Fibro
		IVS9	c.1236-405C>T	p.(Trp412*)				
AT-36	Compd Htz	21 IVS53	c.3111delT; c.7928-1G>A	p.[(Ser1037fs*); Lys2643_Lys2671del)]	absent	no ATMp	classic	LCLs
		36	c.5441T>A	p.(Trp1814*)				
AT-38	Compd Htz	61	c.8814_8824del11	p.(Met2938Ilefs14)	absent	no ATMp	classic	LCLs
		63	c.9169T>G	p.(*3056Glyext*28)				
AT-39	Compd Htz	2	c.-30_2816dup41Kb	exons 2-18 duplicated	absent	no ATMp	classic	Fibro
		IVS4	c.331+2T>G	Ab splice (del ex 4)				
AT-46	Compd Htz	10	c.1369C>T	p.(Arg457*)	absent	no ATMp	classic	Fibro
		24	c.3576G>A	p.(Ser1135_Lys1192del58)				
AT-90	Compd Htz	24	c.3576G>A	p.(Ser1135_Lys1192del58)	15%	25-30%	late onset	LCLs
		33	c.4910-3T>A	p.(Asp1637_Val1654del17)				
AT-0205	Compd Htz	24	c.3576G>A	p.(Ser1135_Lys1192del58)	< 10%	no ATMp	classic	LCLs; Fibro
		IVS10	IVS12+1G>T	p.(Cys536fs*)				
AT-8492	Compd Htz	14	c.2250G>A	p.(Glu709_Lys750del42)	absent	no ATMp	mild	Fibro
		55	c.8122G>A	p.(Asp2708Gln)				

**Supplemental table 2**		
Primers for *ATM* cDNA analysis (NM_000051.3)		
	Forward primer	Reverse primer
ATM cDNA analysis (long-range PCR)	Exon 2<br>5'-agtctagtacttaatgatctgcttatctgc	Exon 56<br>5'-ccaccttataagtacagatagttaatttcc
β-actin	5'-atggatgatgatatcgccgcg	5'-ctagaagcatttgcggtggacgatggag
Primers for *ATM* transcripts assays using absolute RT-qPCR (see scheme in figure 2A) (NM_000051.3)		
ATMdexa1-specific (UPL#89)	Exon 3<br>5'-tcggcattcagattccaaac	Exon 53<br>5'-tgcctcaacacttctgacca
ATM specific assay (no *ATMdexa1*) (UPL#2)	Exon 14<br>5'-cagaattattccagaaagccaagt	Exon 15<br>5'-atttctcaaggaaccaattctga
All *ATM* transcripts (UPL#89)	Exon 3<br>5'-gcattcagattccaaacaagg	Exon 4<br>5'-tctcagacattctgtttctttctga
ATMdexa1 insert generation (see scheme in supplemental figure 1; NM_000051.3)		
left amplimer	Exon 3<br>5'-atctagatcggcattcagattcca	Exon 53-4<br>5'- tgaccatctgaggctgatacattt
right amplimer	Exon 4-53<br>5'-atcagcctcagatggtcagaagtgtt	Exon 54<br>5'-gtttagtaattggctggtctgc
Note: reverse primer for left amplimer and forward primer for right amplimer were designed to be partially reverse complementary (underlined).		

**Supplemental table 3.** Calibration curves for the *ATM* transcripts assays					
pMAT plasmid		pMAT plasmid		pGEM- ATMdexa1 plasmid	
Exons		Exons 3-4		Exons 14-15	
# copies	Ct	# copies	Ct	# copies	Ct
1000000	23,03939	1000000	20,54648	1000000	22,3771
500000	24,10065	500000	21,68904	500000	23,4279
100000	26,68008	100000	24,28088	100000	25,8996
50000	27,81206	50000	25,9564	50000	26,8071
10000	30,65036	10000	27,4764	10000	30,9022
5000	32,41321	5000	28,54222	5000	31,6232
1000	36,46217	1000	29,74249	1000	36,0604
